# Supplementary material for: Systematic Review on Efficacy, Effectiveness, and Safety of Pitavastatin in Dyslipidemia in Asia
Source: Healthcare (Basel). 2024 Dec 31;13(1):59. doi: 10.3390/healthcare13010059 (PMC11720254; doi:10.3390/healthcare13010059)
Supplement: Supplementary file 1 [file healthcare-13-00059-s001.zip › healthcare-3371251-Supplementary Materials.pdf]

healthcare

MDPI

Systematic Review

Systematic Review on Efficacy, Effectiveness, and Safety of Pitavastatin in Dyslipidemia

2.1. Searching strategy

Table S1. History of the syntax used for serving trial identification via Pubmed, Cochrane, and Embase databases.

No.	Syntax	Number of results
	**Pubmed**	
1	(dyslipidemia) AND (pitavastatin) AND ((efficacy) OR (effectiveness) OR (safety)) AND (observationalstudy[Filter])	4
2	(dyslipidemia) AND (pitavastatin) AND ((efficacy) OR (effectiveness) OR (safety)) AND (multicenterstudy[Filter] OR observationalstudy[Filter])	54
3	(dyslipidemia) AND (pitavastatin) AND ((efficacy) OR (effectiveness) OR (safety)) AND (clinicaltrial[Filter] OR multicenterstudy[Filter] OR observationalstudy[Filter])	111
4	(pitavastatin) AND (dyslipidemia) AND ((efficacy) OR (effectiveness) OR (safety)) AND (randomizedcontrolledtrial[Filter])	75
5	(pitavastatin) AND (dyslipidemia) AND ((efficacy) OR (effectiveness) OR (safety))	279
6	(pitavastatin) AND ((dyslipidemia) OR (hyperlipidemia) OR (hypercholesterolemia)) AND ((efficacy) OR (effectiveness) OR (safety))	329
	**Cochrane**	
1	(pitavastatin) AND ((dyslipidemia) OR (hyperlipidemia) OR (hypercholesterolemia)) AND ((efficacy) OR (effectiveness) OR (safety))	86
	**Embase**	
1	('pitavastatin') AND (('dyslipidemia') OR ('hyperlipidemia') OR ('hypercholesterolemia')) AND (('efficacy') OR ('effectiveness') OR ('safety'))	722

3.3. Quality assessment

3.3.1. CONSORT 2010 tool for RCT

Table S2. Assessing the quality of RCT based on CONSORT 2010 criteria.

No	Item	Sansanayudh et al. (1)	Saku et al. (2)	Han et al. (3)	Saito et al. (4)	Park et al. (5)	Lee et al. (6)	Moroi et al., (7)	Kurogi et al. (8)	Liu et al. (9)	Yokote et al. (10)	Sasaki et al. (11)	Saito et al. (12)
1	Title, abstract	0.5	1	0.5	1	1	1	0.5	0.5	1	1	1	0.5
2	Context of research and objective	1	1	1	1	1	1	1	1	1	1	1	1
3	Study design	1	1	1	1	1	1	1	1	1	1	1	1
4	Participants	1	1	1	1	1	1	1	1	1	1	1	1
5	Intervention of trial	1	1	1	1	1	1	1	1	1	1	1	1
6	Primary and secondary outcome	1	1	1	1	1	1	1	1	1	1	1	0.5
7	Sample size	1	0	1	1	1	1	1	0	1	1	1	0
8	Generating randomized sequence	1	0.5	0.5	1	1	1	1	0.5	1	0.5	1	0.5
9	Method to conceal allocation	1	1	0	0	0.5	0	1	0	1	0	1	0
10	Implementation	1	0	0	0	0.5	0.5	0.5	0	0	0	1	0
11	Description of blinding	0	0	0	0.5	0	0	0.5	1	0	0	0	0.5
12	Statistical analysis	1	1	1	1	1	1	1	1	1	1	1	1

13	A diagram to summarize the study's flow	0.5	1	1	0.5	1	1	1	0.5	1	0.5	1	1
14	Detail about how recruitment was performed	1	0	0	0	1	1	1	1	1	1	1	0
15	Baseline information	1	1	1	1	1	1	1	1	1	1	1	0.5
16	Determining number of the groups being analyzed	1	1	1	1	1	1	1	1	1	1	1	0.5
17	Main outcome	1	1	1	1	1	1	1	1	1	1	1	1
18	Subgroup or additional analysis	0	1	1	1	0	0	1	1	1	1	0	0
19	Potential harm or unwanted effect	1	1	1	1	1	1	1	1	1	1	1	1
20	Limitations	1	1	1	0	0.5	1	1	1	1	1	1	0
21	Generalisability	1	1	1	0.5	1	1	1	1	1	1	1	0.5
22	Interpretation	1	1	1	1	1	1	1	1	1	1	1	1
23	Trials registration	0	1	1	0	0	0	1	0	1	0	0	0
24	Protocol-related information	0	1	1	0	0.5	0.5	1	0.5	1	0.5	0.5	0.5
25	Sponsor	1	1	1	0	1	1	1	0	0	0	1	0
	**Total score**	20	20.5	20	16.5	20	20	23.5	18	22	18.5	21.5	13
	**Conclusion**	**Good**	**Good**	**Good**	**Moderate**	**Good**	**Good**	**Good**	**Moderate**	**Good**	**Moderate**	**Good**	**Moderate**

3.3.2. STROBE 2014 tool for non-RCT

Table S3. Assessing risk of bias in non-RCT following STROBE 2014 checklist.

No.	Item	Jeong et al. (13)	Koshiyama et al. (14)	Yoshitomi et al. (15)	Yamasaki et al. (16)	Kong et al. (17)
1	Title, abstract	1	1	1	1	1
2	Background and the rationale for implementing the research	1	1	1	1	1
3	Research's aim	1	1	1	1	1
4	Study design	1	1	1	1	1
5	Detail about conducting condition	1	0.5	1	0	0
6	Description of the targeted participant	1	1	1	1	1
7	Defining outcomes, variables	1	1	1	1	1
8	Measurement of interest outcome, source of data	0.5	1	1	1	1
9	Risk of bias	0	0	0	0	0
10	Sample size	1	0	0	0	0
11	Quantitative variables	1	1	1	1	1
12	Statistical analysis	1	1	1	1	1
13	Description of recruiting participants, including specific numbers assigned to particular groups	1	1	0.5	0.5	0.5
14	Demographic data of eligible participants	1	1	0.5	1	1
15	Reporting number of the outcome event	1	1	1	1	1
16	The primary outcome of the studies	1	1	1	1	1
17	Subgroup or additional analysis	1	0	1	0	1
18	A summary of critical findings	1	1	1	1	1
19	Limitations	1	1	1	1	1
20	Interpretation	1	1	1	1	1
21	Generalisability	1	0.5	1	0.5	1
22	Sponsor information	1	0	1	1	1
	Total score	20.5	17	19	17	18.5
	**Conclusion**	**Low**	**Low**	**Low**	**Low**	**Low**

3.4. Methodological assessment

Table S4. The methodological quality of RCT following JBI appraisal tool.

No.	Question	Sansanayudh et al. (1)	Saku et al. (2)	Han et al. (3)	Saito et al. (4)	Park et al. (5)	Lee et al. (6)	Moroi et al., (7)	Kurogi et al. (8)	Liu et al. (9)	Yokote et al. (10)	Sasaki et al. (11)	Saito et al. (12)

1	Was true randomization utilized for assignment of participants to treatment groups?	Yes	Yes	Yes	Yes	Yes	Yes	Yes	Yes	Yes	Yes	Yes	Yes
2	Were treatment groups allocated in concealment?	Yes	Yes	Unclear	Yes	Yes	Unclear	Yes	Unclear	Yes	Yes	Yes	Unclear
3	Were treatment groups identical at baseline characteristic?	Yes	Yes	Yes	Yes	Yes	Yes	Yes	Yes	Yes	Yes	Yes	Yes
4	Were target subjects blind to treatment assignment?	Unclear	Unclear	No	No	No	No	No	No	No	No	No	Yes
5	Were blinding mentioned in those delivering the treatment assignment?	Unclear	Unclear	No	No	No	No	No	No	No	No	No	Yes
6	Were treatment groups treated in the same conditions other than the interest intervention?	Yes	Yes	Yes	Yes	Yes	Yes	Yes	Yes	Yes	Yes	Yes	Yes
7	Were outcome assessors or investigators blind to treatment assignment?	Unclear	Unclear	No	Unclear	Unclear	Unclear	Yes	Unclear	Unclear	Unclear	Unclear	Yes
8	Were treatment groups measured identically in terms of outcomes?	Yes	Yes	Yes	Yes	Yes	Yes	Yes	Yes	Yes	Yes	Yes	Yes
9	Were outcomes measured in a trustworthy and valid way?	Yes	Yes	Yes	Yes	Yes	Yes	Yes	Yes	Yes	Yes	Yes	Yes
10	Was follow up complete and if not, were the discrepancies among groups discussed adequately?	Yes	Yes	Yes	Yes	Yes	Yes	Yes	Yes	Yes	Yes	Yes	Unclear
11	Were the randomized patients analyzed?	Yes	Yes	Yes	Yes	Yes	Yes	Yes	Yes	Yes	Yes	Yes	Yes
12	Was statistical analysis applied adequately?	Yes	Yes	Yes	Yes	Yes	Yes	Yes	Yes	Yes	Yes	Yes	Yes
13	Were the trials properly planned, and were any variations and any deviations from the standard RCT design (individual randomization, parallel groups) taken into consideration in the conduct and analysis of the trial?	Yes	Yes	Yes	Yes	Yes	Yes	Yes	Yes	Yes	Yes	Yes	Yes
	**Total score**	10	10	9	10	10	9	11	9	10	10	10	11
	**Conclusion**	**Low**	**Low**	**Moderate**	**Low**	**Low**	**Moderate**	**Low**	**Moderate**	**Low**	**Low**	**Low**	**Low**

Table S5. Methodological quality of non-RCT following JBI appraisal checklist.

No.	Question	Jeong et al. (13)	Koshiyama et al. (14)	Yoshitomi et al. (15)	Yamasaki et al. (16)	Kong et al. (17)
1	Were the two groups drawn from the identical population and were they similar?	Yes	Yes	Unclear	Yes	Yes
2	Were the exposures measured similarly in order to categorize individuals into exposed and unexposed groups	No	No	Yes	Yes	Yes
3	Was a valid and trustworthy method used to measure the exposure?	Yes	Yes	Yes	Yes	Yes
4	Were confounding factors acknowledged?	Yes	Yes	Yes	Yes	Yes
5	Were methods for handling confounding variables mentioned?	Yes	Unclear	Yes	Yes	Yes
6	Were the groups free of the outcome at the study's beginning (or at the time of exposure)	Yes	Yes	Yes	Yes	Yes

7	Were valid and reliable methods used to measure outcomes?	Yes	Yes	Yes	Yes	Yes
8	Was the follow-up period documented and lengthy enough for results to emerge?	Yes	Yes	Yes	Yes	Yes
9	Was the follow-up complete, and if not, were the causes of the failure to follow up explained and investigated?	Yes	Unclear	Yes	Unclear	No
10	Were methods used to deal with insufficient follow-up recorded?	Unclear	Unclear	Unclear	Unclear	Unclear
11	Was statistical analysis applied adequately?	Yes	Yes	Yes	Yes	Yes
	**Total score**	9	7	9	9	9
	**Conclusion**	Low	Moderate	Low	Low	Low

References

1. Sansanayudh N, Wongwiwatthananukit S, Putwai P, Dhumma-Upakorn R. Comparative efficacy and safety of low-dose pitavastatin versus atorvastatin in patients with hypercholesterolemia. The Annals of pharmacotherapy. 2010;44(3):415-23.
2. Saku K, Zhang B, Noda K. Randomized head-to-head comparison of pitavastatin, atorvastatin, and rosuvastatin for safety and efficacy (quantity and quality of LDL): the PATROL trial. Circulation journal : official journal of the Japanese Circulation Society. 2011;75(6):1493-505.
3. Han KH, Rha SW, Kang HJ, Bae JW, Choi BJ, Choi SY, et al. Evaluation of short-term safety and efficacy of HMG-CoA reductase inhibitors in hypercholesterolemic patients with elevated serum alanine transaminase concentrations: PITCH study (PITavastatin versus atorvastatin to evaluate the effect on patients with hypercholesterolemia and mild to moderate hepatic damage). Journal of clinical lipidology. 2012;6(4):340-51.
4. Saito Y, Yamada N, Teramoto T, Itakura H, Hata Y, Nakaya N, et al. A randomized, double-blind trial comparing the efficacy and safety of pitavastatin versus pravastatin in patients with primary hypercholesterolemia. Atherosclerosis. 2002;162(2):373-9.
5. Park S, Kang HJ, Rim SJ, Ha JW, Oh BH, Chung N, et al. A randomized, open-label study to evaluate the efficacy and safety of pitavastatin compared with simvastatin in Korean patients with hypercholesterolemia. Clinical therapeutics. 2005;27(7):1074-82.
6. Lee SH, Chung N, Kwan J, Kim DI, Kim WH, Kim CJ, et al. Comparison of the efficacy and tolerability of pitavastatin and atorvastatin: an 8-week, multicenter, randomized, open-label, dose-titration study in Korean patients with hypercholesterolemia. Clinical therapeutics. 2007;29(11):2365-73.
7. Moroi M, Nagayama D, Hara F, Saiki A, Shimizu K, Takahashi M, et al. Outcome of pitavastatin versus atorvastatin therapy in patients with hypercholesterolemia at high risk for atherosclerotic cardiovascular disease. International journal of cardiology. 2020;305:139-46.
8. Kurogi K, Sugiyama S, Sakamoto K, Tayama S, Nakamura S, Biwa T, et al. Comparison of pitavastatin with atorvastatin in increasing HDL-cholesterol and adiponectin in patients with dyslipidemia and coronary artery disease: the COMPACT-CAD study. Journal of cardiology. 2013;62(2):87-94.
9. Liu PY, Lin LY, Lin HJ, Hsia CH, Hung YR, Yeh HI, et al. Pitavastatin and Atorvastatin double-blind randomized comPArative study among hiGh-risk patients, including thOse with Type 2 diabetes mellitus, in Taiwan (PAPAGO-T Study). PLoS One. 2013;8(10):e76298.
10. Yokote K, Bujo H, Hanaoka H, Shinomiya M, Mikami K, Miyashita Y, et al. Multicenter collaborative randomized parallel group comparative study of pitavastatin and atorvastatin in Japanese hypercholesterolemic patients: collaborative study on hypercholesterolemia drug intervention and their benefits for atherosclerosis prevention (CHIBA study). Atherosclerosis. 2008;201(2):345-52.
11. Sasaki J, Ikeda Y, Kuribayashi T, Kajiwara K, Biro S, Yamamoto K, et al. A 52-week, randomized, open-label, parallel-group comparison of the tolerability and effects of pitavastatin and atorvastatin on high-density lipoprotein cholesterol levels and glucose

metabolism in Japanese patients with elevated levels of low-density lipoprotein cholesterol and glucose intolerance. Clinical therapeutics. 2008;30(6):1089-101.

12. Saito Y, Yamada N, Teramoto T, Itakura H, Hata Y, Nakaya N, et al. Clinical efficacy of pitavastatin, a new 3-hydroxy-3-methylglutaryl coenzyme A reductase inhibitor, in patients with hyperlipidemia. Dose-finding study using the double-blind, three-group parallel comparison. Arzneimittel-Forschung. 2002;52(4):251-5.
13. Jeong IK, Kim SR. Efficacy and Safety of Pitavastatin in a Real-World Setting: Observational Study Evaluating SaFety in Patient Treated with Pitavastatin in Korea (PROOF Study). Endocrinology and metabolism (Seoul, Korea). 2020;35(4):882-91.
14. Koshiyama H, Taniguchi A, Tanaka K, Kagimoto S, Fujioka Y, Hirata K, et al. Effects of pitavastatin on lipid profiles and high-sensitivity CRP in Japanese subjects with hypercholesterolemia: Kansai Investigation of Statin for Hyperlipidemic Intervention in Metabolism and Endocrinology (KISHIMEN) investigatars. Journal of atherosclerosis and thrombosis. 2008;15(6):345-50.
15. Yoshitomi Y, Ishii T, Kaneki M, Tsujibayashi T, Sakurai S, Nagakura C, et al. Efficacy of a low dose of pitavastatin compared with atorvastatin in primary hyperlipidemia: results of a 12-week, open label study. Journal of atherosclerosis and thrombosis. 2006;13(2):108-13.
16. Yamasaki T, Iwashima Y, Jesmin S, Ohta Y, Kusunoki H, Hayashi S, et al. Comparison of efficacy of intensive versus mild pitavastatin therapy on lipid and inflammation biomarkers in hypertensive patients with dyslipidemia. PLoS One. 2014;9(2):e89057.
17. Kong SH, Koo BK, Moon MK. Efficacy of Moderate Intensity Statins in the Treatment of Dyslipidemia in Korean Patients with Type 2 Diabetes Mellitus. Diabetes & metabolism journal. 2017;41(1):23-30.
